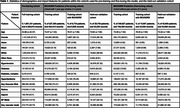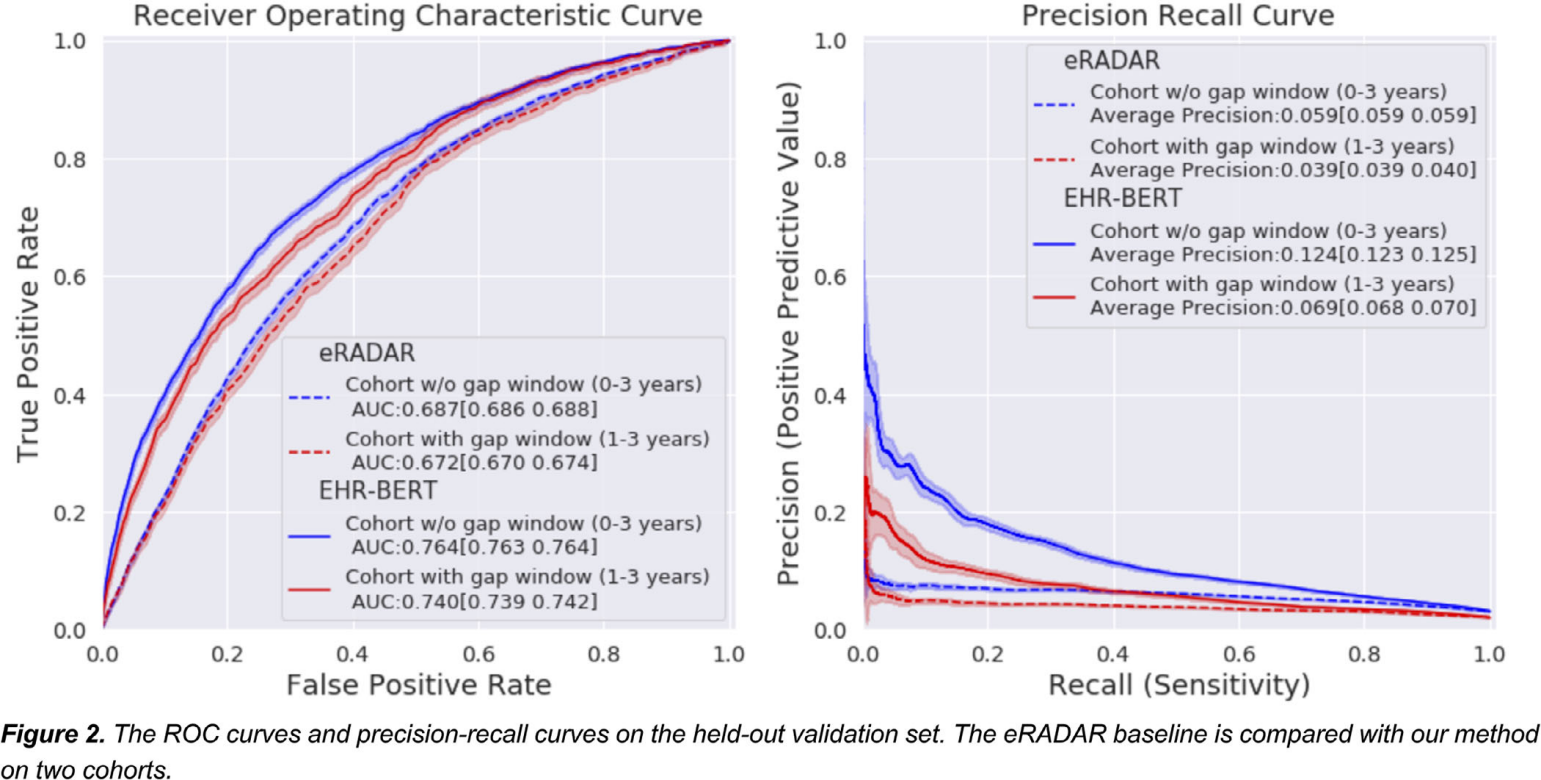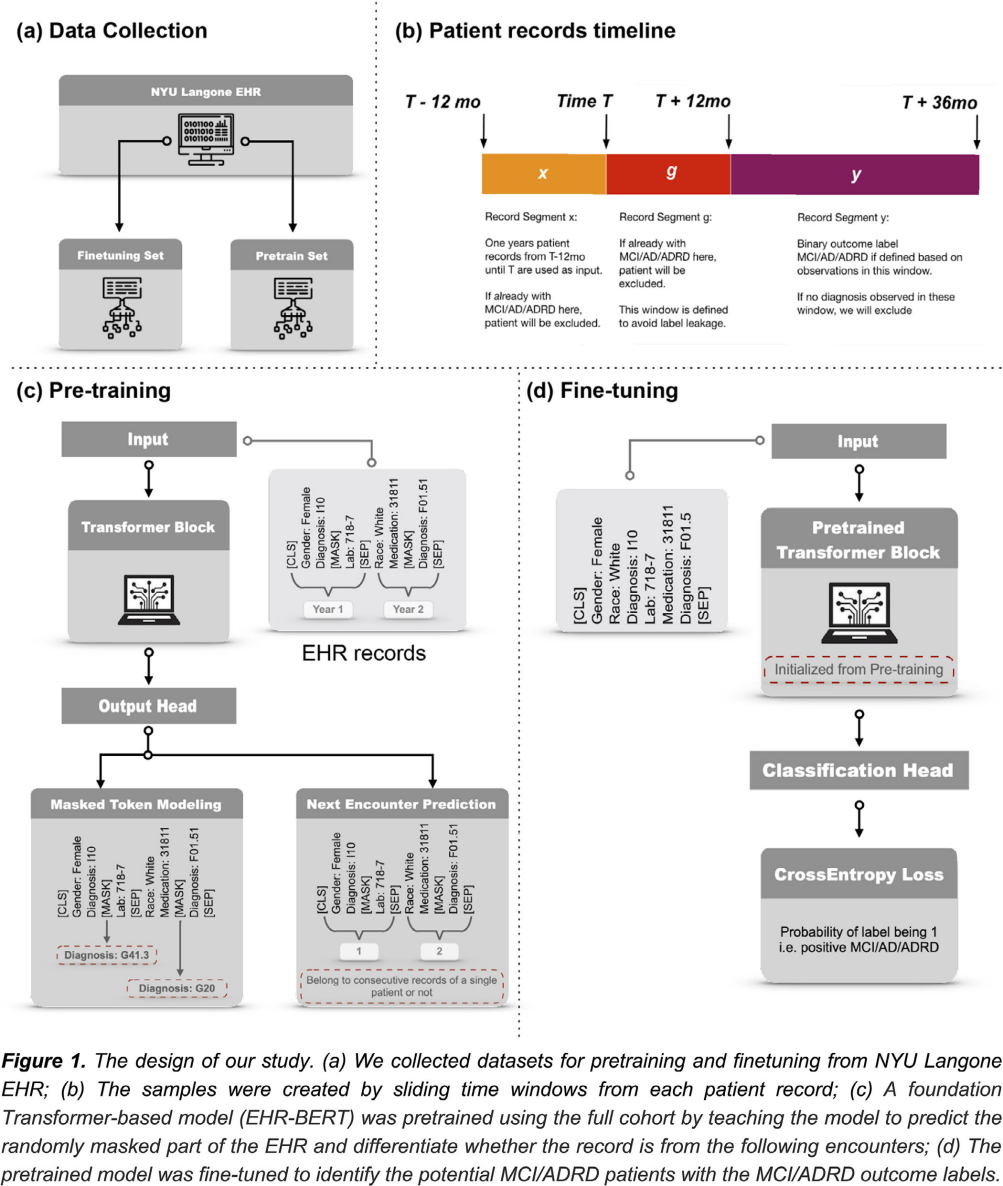# Predicting Alzheimer’s Diseases and Related Dementias in 3‐year timeframe with AI Foundation Model on Electronic Health Records

**DOI:** 10.1002/alz.089281

**Published:** 2025-01-09

**Authors:** Weicheng Zhu, Huanze Tang, Haresh Rengaraj Rajamohan, Divyam Madaan, Ankush Chaudhari, Shih‐Lun Huang, Xinyue Ma, Sumit Chopra, John Dodson, Abraham A. Brody, Arjun V. Masurkar, Narges Razavian

**Affiliations:** ^1^ New York University, New York, NY USA; ^2^ NYU Grossman School of Medicine, New York, NY USA

## Abstract

**Background:**

As disease‐modifying interventions advance, there is a critical need to detect Alzheimer’s disease and related dementias (ADRD) at the earlier, pre‐symptomatic stages. Transformer is a powerful model used to understand high‐dimensional data like images and languages. In this study, we propose a transformer‐based algorithm for predicting mild cognitive impairment (MCI) and ADRD 12 to 36 months in advance based on electronic health records (EHR).

**Method:**

Our study analyzed EHR from NYU Langone between Jan 1 2014 and Jun 29 2022. The patient records were analyzed with sliding windows (Figure 1b): For every index date with at least one‐year lead‐time to onset, records from one preceding year were used as features, and the MCI/ADRD onset within 1‐3 years after the index date served as outcome labels. Patients with MCI/ADRD records in the feature and gap window are excluded to avoid data leakage.

Our prediction framework included two stages ‐ pretraining and finetuning (Figure 1a, c, d). First, we pretrained a foundation Transformer‐based model (EHR‐BERT) using the full cohort, which enabled the model to understand the semantic meanings of variables in EHR. Then we finetuned the pretrained model with the MCI/ADRD outcome labels to identify the potential MCI/ADRD patients. Final predictive performance was evaluated on a fully held‐out validation cohort.

**Result:**

Pretraining used EHRs from 1.98M patients; 947K records from 366K patients over 60, without preexisting MCI/ADRD, were used for finetuning in the downstream prediction task. The demographics are shown in Table 1. We evaluated the prediction performance on a held‐out validation set, consisting of 118,516 records from 46,082 patients. The model obtained AUROC at 0.761 [0.760, 0.762] for prediction in 0‐3 years and 0.740 [0.739, 0.742] in 1‐3 years. Figure 2 shows that EHR‐BERT performed better than existing algorithms in PPVs at the same sensitivity level.

**Conclusion:**

In this study, our transformer‐based AI foundation model, trained on large‐scale electronic health records, demonstrated strong capability in predicting MCI/ADRD up to three years in advance. This algorithm will allow us to advance the current recruitment of dementia screening.